# In adult onset myositis, the presence of interstitial lung disease and myositis specific/associated antibodies are governed by HLA class II haplotype, rather than by myositis subtype

**DOI:** 10.1186/ar1862

**Published:** 2005-12-05

**Authors:** Hector Chinoy, Fiona Salway, Noreen Fertig, Neil Shephard, Brian D Tait, Wendy Thomson, David A Isenberg, Chester V Oddis, Alan J Silman, William ER Ollier, Robert G Cooper

**Affiliations:** 1 Rheumatic Diseases Centre, Hope Hospital, Salford, UK; 2 Centre for Integrated Genomic Medical Research, University of Manchester, Manchester, UK; 3 Division of Rheumatology and Clinical Immunology, University of Pittsburgh School of Medicine, Pittsburgh, PA, USA; 4 arc Epidemiology Research Unit, University of Manchester, Manchester, UK; 5 Victorian Transplantation and Immunogenetic Service, Australian Red Cross Blood Transfusion Service, Melbourne, Australia; 6 Centre for Rheumatology, Department of Medicine, University College London, London, UK

## Abstract

The aim of this study was to investigate HLA class II associations in polymyositis (PM) and dermatomyositis (DM), and to determine how these associations influence clinical and serological differences. DNA samples were obtained from 225 UK Caucasian idiopathic inflammatory myopathy patients (PM = 117, DM = 108) and compared with 537 randomly selected UK Caucasian controls. All cases had also been assessed for the presence of related malignancy and interstitial lung disease (ILD), and a number of myositis-specific/myositis-associated antibodies (MSAs/MAAs). Subjects were genotyped for HLA-DRB1, DQA1 and DQB1. HLA-DRB1*03, DQA1*05 and DQB1*02 were associated with an increased risk for both PM and DM. The HLA-DRB1*03-DQA1*05-DQB1*02 haplotype demonstrated strong association with ILD, irrespective of myositis subtype or presence of anti-aminoacyl-transfer RNA synthetase antibodies. The HLA-DRB1*07-DQA1*02-DQB1*02 haplotype was associated with risk for anti-Mi-2 antibodies, and discriminated PM from DM (odds ratio 0.3, 95% confidence interval 0.1–0.6), even in anti-Mi-2 negative patients. Other MSA/MAAs showed specific associations with other HLA class II haplotypes, irrespective of myositis subtype. There were no genotype, haplotype or serological associations with malignancy. The HLA-DRB1*03-DQA1*05-DQB1*02 haplotype associations appear to not only govern disease susceptibility in Caucasian PM/DM patients, but also phenotypic features common to PM/DM. Though strongly associated with anti-Mi-2 antibodies, the HLA-DRB1*07-DQA1*02-DQB1*02 haplotype shows differential associations with PM/DM disease susceptibility. In conclusion, these findings support the notion that myositis patients with differing myositis serology have different immunogenetic profiles, and that these profiles may define specific myositis subtypes.

## Introduction

The idiopathic inflammatory myopathies (IIMs) are a heterogeneous group of potentially serious diseases, defined by the presence of acquired muscle inflammation and weakness. Polymyositis (PM) and dermatomyositis (DM) are among the most frequently observed subtypes. Although steroids, immunosuppressive agents and intravenous immunoglobulins can all be effective treatments, the therapeutic response to these agents is often disappointing. Thus, PM/DM patients occasionally die from their disease, or as a complication of treatment, while survivors may develop chronic disability through irreversible muscle weakness and/or interstitial lung disease (ILD). Given the relative lack of effectiveness of the available agents for PM/DM, new and more potent therapies are clearly needed. Facilitating the development of such novel therapies would require a better understanding of the aetiopathogenic mechanisms underlying PM/DM, although mechanistic research has proved difficult due to the rarity of these conditions.

Despite such problems, there is increasing evidence that genetic factors are involved in the development of PM/DM [1], although genetically predisposed individuals may only develop their myositis after environmental exposure to specific triggers [1-3]. The rarity of IIMs has precluded concordance studies in twins, but reports of multicase families support a familial predisposition [1]. Candidate gene studies in non-familial IIM have suggested an association of HLA-DRB1*0301 and HLA-DQA1*0501 with IIMs in Caucasians, especially in patients possessing anti-aminoacyl transfer RNA (tRNA) synthetase antibodies and/or ILD [4-6]. These alleles form part of a conserved, ancestral Caucasian haplotype containing A1-B8-Cw7-DRB1*0301-DQA1*0501.

In order to increase statistical power, previous candidate gene IIM studies have typically combined patients with PM and DM, also including those with inclusion body myositis [1]; however, PM and DM differ considerably with respect to their clinical presentations. Thus the classic rashes pathognomic for DM do not occur as part of the PM syndrome, while the association of myositis with malignancy appears considerably stronger for DM than for PM [7]. Immunopathological differences are well documented [8], while differences have also been demonstrated in circulating myositis-specific/myositis-associated antibody (MSA/MAA) profiles [4]. Most patients possessing anti-signal recognition particle antibody (SRP) have PM, whereas an antibody against part of the nucleosome remodelling and deacetylase complex (i.e. the anti-Mi-2 antibody) has high specificity for DM.

It is thus unclear whether PM and DM have a similar genetic susceptibility. Given the differences clearly apparent between the clinical, serological and pathological features of PM and DM, it would seem more appropriate to stratify the patients in any case control study by IIM subtype. We therefore test the hypothesis that HLA class II associations differ between PM and DM, and investigate the contribution of serological profiles to any differences observed.

## Materials and methods

### Design

A cross-sectional, case-control study comparing HLA class II in cases of PM and DM with normal subjects. Subgroup analyses were also undertaken after stratifying by the presence or absence of key MSAs/MAAs.

### Cases

Between 1999 and 2004, a UK-wide group comprising 55 rheumatologists and 4 neurologists (the Adult Onset Myositis Immunogenetic Collaboration (AOMIC), see Acknowledgements) recruited 225 UK Caucasian patients aged 18 years of age or older with probable or definite PM/DM, based on the Bohan and Peter criteria [9,10]. A standardised clinical data collection form, detailing demographics and individual clinical details, was used. The collaborating physicians at each study site confirmed the presence of ILD, by pulmonary function testing and thoracic imaging, and cancer-associated myositis (in the opinion of the recruiting physician), by relevant investigations. Collection of blood from patients was undertaken under regulations of the local research ethics committees.

### Controls

Caucasian control subjects (537) from two sources were recruited: 347 normal subjects recruited from primary population registers in Norfolk, UK, as part of previously described epidemiological studies [11,12] and 260 representing a cohort of UK blood donors collected as controls for other disease studies [13]. Analysis of HLA genotype frequencies between these two sources revealed no differences (data not shown) and thus they were pooled for the current analysis. These subjects' HLA profiles were comparable to well-documented known allelic frequencies for UK Caucasians [14].

### Serological typing

Serum was obtained from 105 PM and 101 DM patients for determination of MSAs/MAAs. Anti-PM-Scl, anti-Mi-2, anti-Ku, anti-U3RNP, anti-U1RNP, anti-SRP, and the anti-tRNA synthetases (anti-Jo-1, anti-PL-7, anti-PL-12, anti-EJ, anti-OJ, and anti-KS) were all determined in a single laboratory by protein immunoprecipitation of the appropriately sized antigen, as previously published [15]. For anti-Ku and anti-Mi-2, immunoprecipitation of the appropriately sized proteins was considered sufficient for determination of the presence of the antibody. The presence of anti-SRP, anti-U3RNP and the rare anti-tRNA synthetases (anti-PL-7, anti-PL-12, anti-EJ, anti-OJ, anti-KS) were confirmed by RNA immunoprecipitation of the appropriately sized RNAs or tRNAS [16]. Anti-PM-Scl, anti-Jo-1 and anti-U1RNP were confirmed by immunodiffusion [17].

### HLA typing

DNA was extracted from a peripheral blood sample obtained from both cases and controls using a standard phenol-chloroform method. Cases were broad-typed for the HLA-DRB1 and DQB1 loci using a commercially available PCR sequence specific oligonucleotide probe typing system (Dynal Biotech GmbH, Hamburg, Germany). All 537 controls were HLA-DRB1 typed, while 153 were HLA-DQB1 typed. The HLA-DQA1 status for patients and 142 controls were derived from the DRB1 and DQB1 results, using well-documented Caucasian haplotype tables [14].

### Statistical analyses

Chi squared tables were used to compare the overall allelic distributions between the myositis subtypes with controls, and exact probabilities calculated using the CLUMP program [18]. Individual HLA phenotypic associations were derived from 2 × 2 contingency tables. Probabilities were calculated using Fisher's exact test and corrected for multiple comparisons using the Bonferroni correction, by multiplying the uncorrected *p* value by the number of alleles tested (12 for DRB1, 6 for DQA1, 5 for DQB1). Data were expressed as odds ratios (ORs) with exact 95% confidence intervals (CIs). ORs were calculated according to Woolf's method with Haldane's correction when critical entries were zero. Linkage disequilibrium (LD) was calculated using 2LD [19]. A forwards and backwards stepwise multivariate logistic regression analysis was also undertaken to determine whether the observed univariate associations were independent of each other [20]. The analyses were also repeated after stratification for myositis serology and the presence of ILD. As strong LD exists across the MHC class II region, DRB1-DQA1-DQB1 haplotypes were assigned to individuals where data for all three loci were available. Haplotypes were estimated for selected loci using the Expectation/Maximization algorithm, as implemented in HelixTree (version 3.1.2, Golden Helix Inc., Bozeman, MT, USA). Unless otherwise stated, the statistical package Stata (release 8, Stata Corp., College Station, TX, USA) was used to perform statistical analysis.

## Results

### Demography

Of the 225 UK Caucasian myositis patients recruited, 117 had PM (81 females, 69.2%), and 108 DM (75 females, 69.4%) (Table 1), confirming the expected female predominance in both myositis subtypes. As shown, the mean age at onset of myositis was similar for PM and DM, at 50.4 versus 49 years, respectively. The median duration of disease at data capture was three years for PM and DM. A similar proportion of patients in each group had ILD (PM = 15.4%, DM = 17.6%). The presence of malignancy was observed in an increased proportion of DM (13.0%) compared to PM (1.7%) patients.

### Overall allelic results

There were large and highly significant differences in overall allelic distributions between PM and controls for the HLA-DRB1 and DQA1 loci (Table 2; *p *= 0.0001). Significant but weaker association was observed between DM and controls at HLA-DRB1 (*p *= 0.009) and DQA1 (*p *= 0.02), but the HLA-DQB1 distribution was more significant in DM (*p *= 0.008) than in PM (*p *= 0.02). These associations were largely accounted for by differences versus controls at the specific alleles: HLA-DRB1*03, DQA1*05 and DQB1*02. In light of this, a 'relative predispositional effect' test was performed to examine whether the effect of other alleles had been masked by the relatively increased frequency of these alleles [21]. HLA-DRB1*03, DQA1*05 and DQB1*02 were therefore removed from the data, and the overall exact tests recalculated, after which no further overall differences were detected between myositis subtype versus controls. When PM was compared directly with DM, significant overall differences were observed at both HLA-DRB1 (*p *= 0.004) and DQA1 (*p *= 8 × 10^-5^).

### HLA associations

As outlined, there were significant increases in the frequencies of HLA-DRB1*03, DQA1*05 and DQB1*02 in PM versus controls (Table 2). In DM versus controls, the frequencies of HLA-DRB1*03 and DQA1*05 were also increased, but to a lesser degree. The frequency of HLA-DRB1*07 was clearly reduced in PM, both compared to controls and DM. The HLA-DQA1*02 results closely mirrored the DRB1*07 results for PM/DM patients and controls. In PM and to a lesser degree DM, both HLA-DRB1*03 and DQA1*05 demonstrated positive and highly significant associations versus controls (Table 3). HLA-DQB1*02 was a risk factor for PM and DM, with a similar strength of association. HLA-DRB1*07 and DQA1*02 were protective factors for PM and, by contrast, were risk factors for DM. Strong pairwise LD was demonstrated between HLA-DRB1*03, DQA1*05 and DQB1*02, and also between DRB1*07 and DQA1*02 (data not shown, *p *< 0.00001). Homozygosity for HLA-DQA1*05 was a risk factor for PM (34.9% versus 9.4%, OR 5.2, 95% CI 1.9–14.8, corrected probability (p_corr_) = 0.003), but conferring no additional risk over DQA1*05 heterozygotes. No further statistical associations with homozygosity were found.

To determine whether there were independent effects in the HLA class II association for PM/DM, a logistic regression model incorporating HLA-DRB1*03, DQA1*05 and DQB1*02 was investigated. In PM, HLA-DQA1*05 had the strongest effect and there was no additional independent effect of DRB1*03 and DQB1*02. For DM, the strongest risk factor was DQB1*02, after accounting for DQA1*05 and DRB1*03. This was confirmed using forwards and backwards stepwise logistic regression. When DM and PM were directly compared, using logistic regression to allow for all other significant alleles, a highly significant between-subtype difference was found due to HLA-DRB1*07 and DQA1*02 (OR 4.2, 95% CI 1.9–9.3 for both).

### Serological subsets

Five DM patients had more than one MSA/MAA, including one with three antibodies (Jo-1, Ku, U1-RNP). In all but one patient (who was Jo-1 and PL-12 positive), the second antibody was anti-U1-RNP. In patients with single MSAs/MAAs, the anti-tRNA synthetase antibodies were the most abundant and detectable in 25% of both PM and DM patients tested (Table 1). Anti-Jo-1 antibody was the most common anti-tRNA synthetase detected. A decreased proportion of patients had negative serology in DM compared to PM (*p *= 0.05), but this was largely attributable to the excess of anti-Mi-2 antibodies observed in DM (16.8% DM versus 1% PM, OR 21.0, 95% CI 3.1–887.7, *p *= 2.9 × 10^-5^). The frequency of anti-SRP antibodies was increased in PM (4.8%) versus DM (2.0%).

In PM/DM combined, HLA-DRB1*03, DQA1*05 and DQB1*02 were all strong risk factors for the presence of anti-tRNA synthetase and anti-PM-Scl antibodies versus controls (Table 4). The associations persisted after stratifying for anti-Jo-1 antibody or myositis subtype. No significant HLA differences were observed between PM and DM in anti-tRNA synthetase positive patients. HLA-DRB1*07, DQA1*02 and DQB1*02 were all strong risk factors in anti-Mi-2-positive patients versus controls. Using logistic regression, HLA-DRB1*07 and DQA1*02 were the main risk factors for the presence of anti-Mi-2 antibodies (data not shown). There were no independent genetic or serological associations observed in the cancer-associated myositis patients (hence these patients were retained in the PM/DM subgroup analysis).

### Haplotype frequencies

LD existed between the HLA class II loci, and thus haplotype frequencies were compared in cases and controls (Table 5). As expected, there was an excess of the DRB1*03-DQA1*05-DQB1*02 haplotype in PM/DM combined versus controls. When stratified by IIM subtype, only the PM versus control association was significant after correction for multiple comparisons. The DRB1*03-DQA1*05-DQB1*02 association was even stronger in anti-tRNA synthetase positive patients versus controls. Compared to controls, the DRB1*07-DQA1*02-DQB1*02 haplotype frequency was increased in DM (*p *= not significant (NS)), but reduced in PM (p_uncorr _= 0.03). The DRB1*07-DQA1*02-DQB1*02 haplotype was a significant risk factor in anti-Mi-2 positive patients versus controls. This haplotype discriminated PM from DM (OR 0.3, 95% CI 0.1–0.6, p_corr _= 0.002), even after allowing for the presence of anti-Mi-2 antibodies (p_uncorr _= 0.03). In patients with no detected antibodies, the DRB1*04-DQA1*03-DQB1*03 haplotype frequency was decreased in PM (16.7%) compared to DM (26.1%).

Examining other antibody associations, the DRB1*03-DQA1*05-DQB1*02 haplotype was also associated with risk for the presence of anti-PM-Scl antibodies, with all 11 anti-PM-Scl positive patients possessing at least one copy. The DRB1*04-DQA1*03-DQB1*03 haplotype frequency was increased in anti-U1-RNP positive patients versus controls (*p *= NS). Both DRB1*02-DQA1*01-DQB1*06 and DRB1*11-DQA1*05-DQB1*03 haplotypes were increased in anti-SRP positive patients versus controls (*p *= NS for both).

### Interstitial lung disease

There was a strong association of anti-tRNA synthetase positive patients with ILD (OR 9.5, 95% CI 3.9–23.9, *p *= 2 × 10^-09^), irrespective of myositis subtype. A striking observation was that 21/22 patients with ILD in association with an anti-tRNA synthetase possessed at least one copy of HLA-DRB1*03-DQA1*05-DQB1*02 (haplotype frequency 52.3% disease versus 16.5% controls, OR 5.5, 95% CI 2.6–11.6, p_corr _= 1 × 10^-05^). Of the remaining ILD group with a detected antibody, four possessed anti-PM-Scl and one possessed anti-SRP antibodies.

As HLA-DQB1*02 could be shared between the HLA-DRB1*03-DQA1*05-DQB1*02 and DRB1*07-DQA1*02-DQB1*02 haplotypes, we examined patients with both haplotypes. Twelve patients possessed HLA-DRB1*03/*07, DQA1*05/*02 and at least one copy of DQB1*02; 50% of these patients also had ILD. Of all the patients with the HLA-DRB1*07-DQA1*02-DQB1*02 haplotype, however, none had ILD unless DRB1*03 and DQA1*05 were also present. Possessing both haplotypes was negatively associated with development of anti-Mi-2 antibodies, and no such patients possessed ILD either. Of note, three anti-Mi-2 positive patients possessed a copy of HLA-DRB1*03 and DQA1*05, a finding that has not previously been described.

## Discussion

The results from this study confirm the previously reported influence of HLA class II associations in governing PM/DM disease susceptibility in Caucasians [4,5,22]. However, the current results also demonstrate important differences between PM and DM, in the relative strengths of their HLA class II associations, and in their contrasting associations with HLA-DRB1*07 and DQA1*02. Furthermore, the observed haplotypes appear to influence clinical features in PM/DM, including the presence or absence of ILD, and the pattern of circulating MSAs/MAAs detected. Thus, HLA class II associations appear to not only govern disease susceptibility in PM and DM, but also to govern the expression of certain phenotypic features common to both myositis subtypes.

The PM/DM subtype differences detected may be partly explained by their differing serological associations. Thus, HLA-DRB1*07 and DQA1*02 are risk factors for DM and anti-Mi-2 antibodies, whereas anti-Mi-2 is rare in PM, where these alleles are protective. However, HLA-DRB1*07 and DQA1*02 still discriminate between PM and DM even after allowing for the presence of anti-Mi-2 antibodies. In PM, it is possible that the high DRB1*03-DQA1*05-DQB1*02 frequency may be responsible for lowering the DRB1*07-DQA1*02-DQB1*02 frequency, due to the shared DQB1*02 allele. Indeed, in DM, HLA-DQB1*02 had the strongest effect because of the increased frequency of both haplotypes. The DRB1*03-DQA1*05-DQB1*02 haplotype is associated with anti-tRNA synthetases, and the development of ILD in patients of both myositis subtypes. The negative associations of HLA-DRB1*07-DQA1*02-DQB1*02 and anti-Mi-2 antibodies with ILD suggest a genetically determined patient cohort with a favourable outcome. The strong associations of DRB1*03-DQA1*05-DQB1*02 with anti-synthetases and ILD suggest a genetically determined patient cohort with an unfavourable outcome.

Moreover, the presence of HLA-DRB1*03 and DQA1*05 appear to render HLA-DRB1*07-DQA1*02-DQB1*02 positive patients susceptible to ILD. Therefore, at PM/DM disease outset, knowledge of haplotype and anti-tRNA synthetase/Mi-2 antibody status could potentially improve outcome in respect of ILD detection and also help physicians make informed choices regarding use of agents capable of inducing lung fibrosis. There are methodological issues that require discussion. As patient recruitment was multi-centre, disease subtype misclassification of a small number of patients is a possibility; however, this should have reduced the likelihood of finding subtype differences and would, if anything, have made the results more conservative. Misclassification may also explain the PM anti-Mi-2 and DM anti-SRP positive patients, although these MSAs are not thought to be as disease specific as previously thought [16,22]. A cross-sectional study design was used for patient recruitment, and this may have resulted in underestimation of ILD and malignancy. We were able to type most of the MSAs and MAAs associated with myositis, but some antibodies were not tested for (for example, anti-Ro52, the antibody against tertiary tRNA (anti-WS), and anti-translation factor (anti-KJ)), which may partly explain some of the genotypic association differences between PM and DM and results observed in patients where none of the tested antibodies were detected.

The term haplotype describes a set of closely linked alleles present on one chromosome that are inherited together. Certain combinations of alleles for HLA loci are also found in strong LD, referred to as conserved or ancestral haplotypes. Clearly, if clinical disease features are associated with these haplotypes, such features could be retained over time within a population. Love *et al*. [4] suggested that IIMs should be classified according to their serological subsets, and that the respective antibodies could be broadly defined by their HLA associations. Our data builds on recent findings [23,24] suggesting that this statement can be broadened to include not only allelic, but multiple haplotypic associations. It is an intriguing serological characteristic of PM/DM that anti-tRNA synthetase antibodies and other highly specific autoimmune responses are generated against components of the intracellular translational machinery. Possession of a specific haplotype within the MHC molecule may make processing and presentation of such intracellular components more likely [25]. The conserved HLA-DRB1*03-DQA1*05-DQB1*02 haplotype likely represents, or is a marker for, a true disease susceptibility gene in PM and DM. However, due to very strong LD shared within the haplotype, and the limited examination of the region to date in IIMs, it is currently difficult to speculate further about the precise location of such a gene.

It is also interesting to note the observation of a latitudinal effect on myositis disease expression [26-28], where patients with DM, as a proportion of those patients with PM or DM, correlated with natural UV radiation, as did possession of the anti-Mi-2 antibody [28]. HLA-DR7, which is associated with DM and with anti-Mi-2 antibodies, has also been associated with non-melanoma skin cancers in both immunocompetent [29] and immunosuppressed renal transplant patients [30], and is a protective factor for skin cancer in heart transplant patients [31]. Also, the B14-Cw6-DR7 and B57-Cw6-DR7 haplotypes are associated with psoriasis [32]. We speculate these findings may reflect a polymorphism on the DRB1*07 haplotype responsible for epidermal cell development, which could influence cutaneous disease expression in myositis.

## Conclusion

One of AOMIC's major objectives was to provide myositis subtype cohort sizes sufficiently large and statistically powerful to compare IIM subtypes. Of greater potential importance, however, is the confirmation that PM and DM possess HLA class II haplotype associations, and that genetic differences observed between PM and DM can be partly accounted for by their serological differences. Myositis disease subtypes appear to be defined by specific haplotypes acting as risk factors for the development of various MSAs and MAAs. It is hoped that, during future PM/DM genetic comparisons, enough statistical power will be present to produce results of sufficient quality to improve our understanding of the aetiological mechanisms underlying these diseases.

## Abbreviations

AOMIC = Adult Onset Myositis Immunogenetic Collaboration; CI = confidence interval; DM = dermatomyositis; IIM = idiopathic inflammatory myopathy; ILD = interstitial lung disease; LD = linkage disequilibrium; MAA = myositis-associated antibody; MSA = myositis-specific antibody; NS = not significant; OR = odds ratio; p_corr _= corrected probability; PM = polymyositis; SRP = signal recognition particle; tRNA = transfer RNA.

AOMIC = Adult Onset Myositis Immunogenetic Collaboration; CI = confidence interval; DM = dermatomyositis; IIM = idiopathic inflammatory myopathy; ILD = interstitial lung disease; LD = linkage disequilibrium; MAA = myositis-associated antibody; MSA = myositis-specific antibody; NS = not significant; OR = odds ratio; p_corr _= corrected probability; PM = polymyositis; SRP = signal recognition particle; tRNA = transfer RNA.

## Competing interests

The authors declare that they have no competing interests.

## Authors' contributions

HC performed the analysis and drafted and revised the manuscript. FS carried out the genotyping. NF carried out the serological typing. NS assisted with the statistical analysis. BT assisted with the genotyping. WT assisted with the genotyping and contributed to preparation of the manuscript. DI helped with setting up AOMIC. CO oversaw the serological typing and contributed to preparation of the manuscript. AS helped to prepare the manuscript. WO oversaw the genotyping, contributed to interpretation of the findings and preparation of the manuscript. RC set up AOMIC, oversaw the whole project and helped to prepare the manuscript. All authors read and approved the manuscript.

## Figures and Tables

**Table 1:** Patient details and antibody frequencies

	n (%)	
	Polymyositis	Dermatomyositis

	(n = 117)	(n = 108)

Females	81 (69.2)	75 (69.4)
Average age of onset^a^	50.4 ± 14.5	49.0 ± 14.1
Interstitial lung disease	18 (15.4)	19 (17.6)
Malignancy^b^	2 (1.7)	14 (13.0)
Antibody status	(n = 105)	(n = 101)
Myositis-specific antibodies		
Jo-1	24 (22.9)	22 (21.8)
PL-7	1 (1.0)	0
PL-12	0	1 (1.0)
EJ	0	1 (1.0)
OJ	1 (1.0)	1 (1.0)
KS	1 (1.0)	1 (1.0)
Any of the above^c^	27 (25.7)	25 (24.7)
Mi-2^d^	1 (1.0)	17 (16.8)
SRP	5 (4.8)	2 (2.0)
Myositis-associated antibodies		
U1-RNP	5 (4.8)	8 (7.9)
U3-RNP	0	2 (2.0)
Ku	0	2 (2.0)
PM-Scl	5 (4.8)	6 (5.9)
None of the above autoantibodies	62 (59.1)	45 (44.5)

^a^Results expressed as mean ± standard deviation. ^b^Dermatomyositis (DM) versus polymyositis (PM), *p *= 0.001; odds ratio (OR) 8.6, (95% confidence interval (CI) 1.9–78.9). ^c^The total for DM is 25 despite the presence of 26 anti-tRNA synthetases, due to one patient possessing both anti-Jo-1 and anti-PL-12. ^d^DM versus PM, *p *= 2.9 × 10^-5^; OR 21.0 (95% CI 3.1–887.7).

**Table 2:** Frequency of HLA class II phenotypes

HLA	Controls	Polymyositis	Dermatomyositis
	n (%)	n (%)	n (%)

DRB1	(n = 537)	(n = 115)	(n = 107)
01	127 (23.6)	25 (21.7)	27 (25.2)
02	145 (27.0)	23 (20.0)	19 (17.8)
03	151 (28.1)	72 (62.6)	50 (46.7)
04	195 (36.3)	31 (27.0)	39 (36.4)
07	129 (24.0)	11 (9.6)	37 (34.6)
08	37 (6.9)	5 (4.3)	2 (1.9)
09	12 (2.2)	1 (0.9)	2 (1.9)
10	8 (1.5)	3 (2.6)	0 (0)
11	61 (11.4)	16 (13.9)	11 (10.3)
12	11 (2.0)	3 (2.6)	1 (0.9)
13	96 (17.9)	18 (15.6)	10 (9.3)
14	30 (5.6)	5 (4.3)	5 (4.7)
p		0.0001	0.009
DQA1	(n = 142)	(n = 110)	(n = 104)
01	92 (64.8)	62 (56.4)	57 (54.8)
02	33 (23.2)	10 (9.1)	36 (34.6)
03	60 (42.3)	31 (28.2)	39 (37.5)
04	5 (3.5)	3 (2.7)	1 (1.0)
05	55 (38.7)	82 (74.5)	58 (55.8)
06	2 (1.4)	2 (1.8)	0 (0)
p		0.0001	0.02
DQB1	(n = 153)	(n = 116)	(n = 108)
02	61 (39.9)	76 (65.5)	71 (65.7)
03	87 (56.9)	56 (48.3)	57 (52.8)
04	9 (5.9)	5 (4.3)	3 (2.8)
05	42 (27.5)	35 (30.2)	32 (29.6)
06	65 (42.5)	37 (31.9)	31 (28.7)
p		0.02	0.008

*P*, global probability for disease versus controls (using genotype data). n (%), number/percentage of patients with individual phenotypes.

**Table 3:** Results of univariate analyses for disease versus controls

	Polymyositis			Dermatomyositis		
HLA phenotype	*p*	*p*_corr_	OR (95% CI)	*p*	*p*_corr_	OR (95% CI)

DRB1*03	6 × 10^-12^	7 × 10^-11^	4.3 (2.8–6.7)	2 × 10^-04^	0.003	2.2 (1.4–3.5)
DRB1*07	4 × 10^-04^	0.005	0.3 (0.2–0.6)	0.03	NS	1.7 (1.04–2.6)
DQA1*02	0.004	0.02	0.3 (0.1–0.7)	0.06	NS	1.7 (0.96–3.2)
DQA1*05	1 × 10^-08^	9 × 10^-08^	4.6 (2.6–8.3)	0.01	0.06	2.0 (1.2–3.4)
DQB1*02	4 × 10^-05^	2 × 10^-04^	2.9 (1.7–4.9)	5 × 10^-05^	3 × 10^-04^	2.9 (1.7–5.0)

CI, confidence interval; NS, not significant; OR, odds ratio; *p*, probability; *p*_corr_, corrected probability.

**Table 4:** Comparison of HLA class II phenotypes in serological subsets^a^

HLA phenotype/serology	n (%)	*p*	*p*_corr_	OR (95% CI)
DRB1*03				
Synthetase	44 (84.6)	1 × 10^-15^	1 × 10^-14^	14.1 (6.3–35.2)
PM-Scl	11 (100)	3 × 10^-6^	4 × 10^-5^	30.6 (4.4–1309.1)
DRB1*07				
Mi-2	14 (77.8)	4 × 10^-6^	5 × 10^-5^	11.1 (3.4–46.8)
DQA1*02				
Mi-2	14 (77.8)	9 × 10^-6^	5 × 10^-5^	11.6 (3.3–50.6)
DQA1*05				
Synthetase	42 (85.7)	7 × 10^-9^	4 × 10^-8^	9.5 (3.8–26.5)
PM-Scl	11 (100)	0.0002	0.001	18.9 (2.6–814.9)
DQB1*02				
Synthetase	42 (85.7)	6.6 × 10^-9^	4 × 10^-8^	9.5 (3.8–26.5)
Mi-2	15 (83.3)	7 × 10^-4^	0.004	7.5 (2.0–41.9)
PM-Scl	11 (100)	0.0003	0.001	18.0 (2.5–777.4)

^a^Results are versus controls. CI, confidence interval; OR, odds ratio; *p*, probability; *p*_corr_, corrected probability.

**Table 5:** Estimated haplotype frequencies of HLA class II loci

DRB1-DQA1-DQB1 haplotype	%							
	Controls	PM	DM		Other antibodies^a^			

		Overall	Overall	AS	Mi-2	PM-Scl	U1-RNP	SRP

	2n = 284	2n = 220	2n = 208	2n = 98	2n = 36	2n = 22	2n = 24	2n = 12

04-03-03	20.4	16.4	19.1	17.3	13.8	4.5	37.5	0
03-05-02^b^	16.5	33.6	24.5	43.9	8.3	54.5	12.5	4.1
02-01-06	13.7	9.1	9.6	10.2	8.3	4.5	20.8	25.0
01-01-05	10.6	11.8	13.5	7.1	22.2	9.1	16.7	8.3
13-01-06	10.2	6.4	5.8	6.1	0	4.5	1.0	8.3
07-02-02^c^	9.2	4.1	13.9	7.1	33.3	18.2	0	0
11-05-03	4.6	7.3	5.3	2.0	5.6	0	0	16.7
07-02-03	3.9	0.4	3.8	0	5.6	0	1.0	0

Probabilities stated are corrected for multiple comparisons. Haplotypes found in less than 3% of controls are excluded from the table. ^a^Polymyositis and dermatomyositis patients combined. ^b^PM versus controls, *p *= 1.1 × 10^-4^, odds ratio (OR) 2.6 (95% confidence interval (CI) 1.6–4.0); AS versus controls, *p *= 7 × 10^-10^, OR 4.8 (CI 2.8–8.3); PM-Scl versus controls, *p *= 0.001, OR 6.1 (CI 2.2–16.5). ^c^PM versus DM, *p *= 0.004, OR 0.3 (CI 0.1–0.6); Mi-2 versus controls, *p *= 0.002, OR 4.9 (CI 2.0–11.6). AS, anti-tRNA synthetase positive; DM, dermatomyositis; PM, polymyositis.
